# Identifying Human Kinase-Specific Protein Phosphorylation Sites by Integrating Heterogeneous Information from Various Sources

**DOI:** 10.1371/journal.pone.0015411

**Published:** 2010-11-15

**Authors:** Tingting Li, Pufeng Du, Nanfang Xu

**Affiliations:** 1 Department of Biomedical Informatics, Peking University Health Science Center, Beijing, China; 2 Ministry of Education Key Laboratory of Bioinformatics and Bioinformatics Division, Tsinghua National Laboratory for Information Science and Technology/Department of Automation, Tsinghua University, Beijing, China; 3 School of Computer Science and Technology, Tianjin University, Tianjin, China; 4 Peking University First Hospital, Beijing, China; Indiana University School of Medicine, United States of America

## Abstract

Phosphorylation is an important type of protein post-translational modification. Identification of possible phosphorylation sites of a protein is important for understanding its functions. Unbiased screening for phosphorylation sites by *in vitro* or *in vivo* experiments is time consuming and expensive; *in silico* prediction can provide functional candidates and help narrow down the experimental efforts. Most of the existing prediction algorithms take only the polypeptide sequence around the phosphorylation sites into consideration. However, protein phosphorylation is a very complex biological process *in vivo*. The polypeptide sequences around the potential sites are not sufficient to determine the phosphorylation status of those residues. In the current work, we integrated various data sources such as protein functional domains, protein subcellular location and protein-protein interactions, along with the polypeptide sequences to predict protein phosphorylation sites. The heterogeneous information significantly boosted the prediction accuracy for some kinase families. To demonstrate potential application of our method, we scanned a set of human proteins and predicted putative phosphorylation sites for Cyclin-dependent kinases, Casein kinase 2, Glycogen synthase kinase 3, Mitogen-activated protein kinases, protein kinase A, and protein kinase C families (avaiable at http://cmbi.bjmu.edu.cn/huphospho). The predicted phosphorylation sites can serve as candidates for further experimental validation. Our strategy may also be applicable for the *in silico* identification of other post-translational modification substrates.

## Introduction

Protein phosphorylation is a kind of post-translational modification which plays key roles in many cellular processes. A protein kinase catalyzes the protein phosphorylation process, in which the γ phosphate on ATP or GTP is transferred to the substrates. Protein phosphorylation has the following characteristics: 1) phosphorylation requires a protein kinase to catalyze the reaction. There are currently 518 known kinase genes in the human genome [1]. These kinases are divided into 134 families according to the sequence of their catalytic domain [1]. 2) Phosphorylation usually takes place on particular amino acids of the substrate protein. In eukaryotic cells, it occurs mainly on Serine (S), Threonine (T) or Tyrosine (Y). 3) The phosphate on substrates can be removed by phosphatases, so the phosphorylation process is reversible: it is determined by the balance between protein kinases and phosphatases.

This reversible character allows the phosphorylation process to work like a switch in a living cell. When there is an external input signal, protein kinases activate specific substrates. After singla wanes, the activated substrates will be “shut down” by phosphatases, the substrates return to their original state and wait for the next signal. A normal biological function *in vivo* usually involves a series of phosphorylation processes [2]. In an eukaryotic cell, about 30–50% of the proteins can be phosphorylated [3]. To regulate so many proteins simultaneously, there must be a mechanism that can control the protein phosphorylation process precisely. Protein kinases play important roles in this mechanism. They recognize specific substrates and determine the exact time and place for phosphorylation to occur. Thus, the identification of the involved kinases and their phosphorylation sites are the first step to understand mechanisms.

^32^p-labeling and mass-spectroscopy are common experimental methods to identify phosphorylation sites, however, both of them are costly and time consuming if applied in an unbiased fashion. Thus, using computational methods to screen for putative sites prior to experimental verification can narrow down the efforts on experimental work. Many computational methods for identifying phosphorylation sites have been developed. In 1998, Kreegipuu *et al.* found that the primary peptide sequences around phosphorylation sites have strong signals for a collection of known phosphorylation sites. These signals can be used to identify possible phosphorylation sites [4]. In 1999, Blom *et al.* utilized the information of the peptide sequences in the proximity of the potential phosphorylation sites to develop the first phosphorylation site prediction method based on an artificial neural network algorithm [5]. After that, many advanced machine-learning algorithms had been introduced to predict the phosphorylation sites, such as logistics regression [6], support vector machine (SVM) [7] and conditional random field [8]. In our previous work, we developed a kinase-specific phosphorylation site prediction algorithm by the log-odds ratio approach based on the peptide sequences surrounding the potential phosphorylation sites [9].

Although most of existing methods predict phosphorylation sites based solely on the primary sequences around the phosphorylation sites, the primary sequences cannot fully determine whether the phosphorylation will occur. There are at least three mechanisms that can affect the phosphorylation process *in vivo* [2]: 1) Kinases interact with amino acids around phosphorylation sites directly. Take kinase PKA as an example, the glutamic acid at position 170 and 230 of PKA kinase can interact with the second arginine downstream of phosphorylation sites on substrates [10]. 2) Protein kinases (e.g. Kinase MAPK) can interact with their substrates through docking sites far away from the phosphorylation sites [11]. 3) Protein kinases (e.g. Kinase PKA) interact with their substrates through an intermediate scaffold protein [12]. Both mechanisms 2) and 3) reduce the dependency of protein phosphorylation on the peptide sequences around the phosphorylation sites. It is even more complicated when considering the higher level structure of the protein. If a peptide sequence can be recognized by a kinase but is buried inside the proteins high-level structures, the kinase still can not interact with it. It remained to be evaluated whether using information in addition to the primary sequence (e.g. subcellular location, functional domains and high-level structures) can increase the prediction accuracy.

Till recently, some efforts have been made in this direction. In 2007, Gnad *et al.* integrated protein secondary structure into phosphorylation site prediction [13]; and Linding *et al.* took the protein-protein interaction information into consideration [14]. Both of them showed increase in predictive power and reduce in false positives. Some other functional features may also be relevant for predicting phosphorylation sites. In our previous study, we found that although one protein kinase can recognize various proteins, these substrates have significant similarity in terms of their biological functions [9]. Take the CDK kinase family as an example: in a GO analysis, terms like DNA binding and transcriptional regulation are enriched in their substrates. This observation is consistent with the previous reports that sequential activation of different kinases in the CDK family regulates DNA replication, cell division and transcription processes [15], [16], [17], [18], [19]. Besides, the subcellular localization of a protein can also affect the phosphorylation process substantially; because kinases and their substrates cannot meet and interact with each other if they are not localized to the same cellular compartment. Several papers have discussed this issue [7], [20], but no one took advantage of the subcellular location information for prediction. Furthermore, the proteins functional domains might also contain some useful information for phosphorylation.

In this study, we try to integrate primary sequences with functional features, including KEGG pathways, GO terms, protein-protein interactions and protein functional domains, to predict phosphorylation sites. The final results indicated that for most of the kinase families, integration of functional features can improve the prediction performance, especially for the GSK3 kinase family, in which we can achieve about 10% improvement in accuracy. Finally, we scanned the human proteome for the kinase-specific phosphorylation sites using this new strategy. These identified putative phosphorylation sites can serve as a set of reliable candidates for experimental validation.

## Methods

### Data preparation

#### Positive training dataset

The phosphorylation sites were extracted from the Phospho.ELM Database (Version 8.2) [21], [22]. This dataset has been used as a benchmarking dataset to evaluate the performance of phosphorylation site prediction methods [23], [24]. Phospho.ELM contains experimentally verified phosphorylation sites of proteins from eukaryotic cells. Version 8.2 contains 4687 protein entries covering 19649 phosphorylation instances. For each entry, it provides information about substrate proteins with the exact positions of the residues that are experimentally verified to be phosphorylated by a given kinase. Since we considered protein functional information in this study, we extracted a dataset containing only human phosphorylation sites containing 11038 entries. For each phosphorylation site, we extracted the 9-mer sequence, including the central residue and the −4 to +4 amino acids surrounding it. The prediction was performed in a kinase-specific way and the known phosphorylation sites of each kinase family/subfamily were extracted separately. The kinase families containing at least 50 experimental phosphorylation sites were used in this study, they are: Ataxia telangiectasia mutated (ATM), Cyclin-dependent kinases (CDK), Casein kinase 2 (CK2), Glycogen synthase kinase 3 (GSK-3), Mitogen-activated protein kinases (MAPK), cAMP-dependent protein kinase (PKA), Protein kinase B (PKB) and Protein kinase C (PKC).

#### Background protein set

The background protein set contains all the human proteins of Swiss-Prot database (version 56.0).

#### Background set and Negative training dataset

It is well known that protein phosphorylation is a dynamic event and depends heavily on conditions [2]. Many sites that are not reported as phosphorylated in one experiment may be phosphorylated in other tissues or conditions. For some other proteins which are currently not reported as phosphorylated, the reason might be that they are not expressed at the same time or in the same tissue with the protein kinase. It is hard to collect a set of protein sequences which can be safely regarded as non-phosphorylatable. The available method usually uses reported phosphorylation sites in phosphorylated proteins as positive samples, and unreported possible phosphorylation sites (usually Serine, Threonine or Tyrosine) in phosphorylated proteins as negative samples. This is not appropriate, since what we usually want is to predict phosphorylation sites for proteins without known phosphorylation sites. So a leaning machine trained based only on the sites of known phosphorylated proteins might have a bias.

To estimate the performance of different kinds of features, we randomly selected the negative dataset from the background set. The background set was constructed from all S/T centered 9-aa peptides extracted from proteins of the background protein set excluding those known as phosphorylation sites. It is true that some unknown phosphorylation sites might be included in the negative dataset. Although phosphorylation occurs very frequently in cells, compared to the large amount of peptide sequences, the amount of phosphorylation sites of a specific kinase is still small. So the non-phosphorylated amino acids for the corresponding kinase family should dominate the negative dataset.

#### Sequence level features

In addition to primary sequences around phosphorylation sites, protein secondary structure has been found to be informative in phosphorylation site prediction [13]. So we also integrated the protein secondary structure and accessibility features. Because the known protein structure information is very limited; we predicted the protein secondary structure and accessibility from the primary protein sequences using SABLE [25]. Since secondary structure and accessibility features were predicted from the primary protein sequences we took both the protein structure and the primary sequences around phosphorylation sites as sequence level features.

#### Functional level features

Besides sequence level features, we aimed to find out functional information which could improve the performance of phosphorylation site prediction. We used following data sources: 1) KEGG pathways which were downloaded from the KEGG database (version 2009/09/16). 2) Biological Process, 3) Molecular Function and 4) Cellular Component annotation files from the GO database (version 1.120). 5) Protein-protein interactions (PPI), which were downloaded from the STRING database (version 8.0). Protein functional domain information were extracted from 6) the Pfam (version 23.0) and 7) InterPro (version 18.0) Databases.

### Over-represented and under-represented feature analysis

Functional data contains huge number of features for each protein. To reduce the dimensionality of feature space, we only used the over-represented or under-represented features for the substrate proteins in each kinase family. Two sided hypergeometric tests were used to detect over-represented or under-represented terms in the study set compared to the background protein set. Here the study set is the substrates of each kinase family. P-values derived from hypergeometric distributions were corrected by Bonferroni correction for testing on multiple terms. Terms with Bonferroni corrected p-values less than 1e-2 were taken as significant. The calculations were performed using the R package for statistical computing [26].

### Scoring the protein

In previous studies, the identification of phosphorylated proteins is usually by the prediction of phosphorylation sites. Here we want to find out whether the phosphorylated proteins can be discriminated from the non-phosphorylated ones based only on the functional features. A simple log-odds ratio approach was used. For binary feature *i* (*i* = 1, 2, …, n, where n is the number of significant functional features for the kinase family), its value *x_i_* for the candidate protein is measured from the functional annotations of the protein. We estimated its probability *f*(*x_i_*) in phosphorylated proteins from the positive training set and its probability *g*(*x_i_*) in all proteins from the background protein set. As described above, the background protein set was constructed from all the human proteins in the Swiss-Prot database. The log-odds score for feature *i* of the candidate protein is defined as the log ratio of the two conditional probabilities:
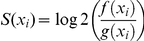



The log-odds for all features can be summed up as the final score for a protein. It measures the log-odds for it belongs to the phosphorylated class versus the background. By summation, we implicitly assumed that all features are independent from each other. But indeed, some of the enriched functional features here should be correlated with each other, for example some annotations in KEGG might be similar to those in GO Biological Process. So we used a modified log-odds ratio score. The central consideration is that the higher the similarity of a feature is to all remaining features, the lower its weight should be. The similarity between two features *r* and *s* was measured by the Jaccard distance, which is equal to the percentage of nonzero coordinates that differ:
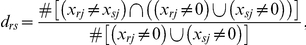
where *j* is the sample size.

For each feature *i*, the weight *w* is the sum of all its similarities to the remaining n−1 features:
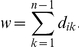
So the contribution of each feature to the final becomes 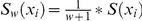.

The final score for each protein is given by 
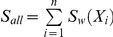
.

### Feature representation

To transform protein sequences into numeric vectors, each amino acid is represented as a 20-bit binary tuple (each bit is an indicator for one of twenty amino acids). For example, serine (S) is expressed as a 20-dimensional vector [0,0,0,0,0,0,0,0,0,0,0,0,0,0,0,0,1,0,0,0]^T^ and threonine (T) is expressed as vector [0,0,0,0,0,0,0,0,0,0,0,0,0,0,0,0,0,1,0,0]^T^. Therefore, if the window size of a candidate sequence is 9, the dimension of the feature vector representing it is 20*9.

There are two kinds of structure-related features: secondary structure and accessibility. For each amino acid of the query sequence, SABLE provides three kinds of secondary structure: (H→helix, E→beta strand, C→coil). Since the phosphorylation sites are preferentially in the coiled sites [6], we divided these three structures into two groups: coil and not-coil. And transform protein structures into binary numbers with both Helix and beta strand represented by 0 and coil represented by 1. For the accessibility features, SABLE provides a score ranged from 0 to 9 (0→fully Buried, 9→fully Exposed). From the prediction results we found that the scores of our candidates ranged from 0 to 6, so we used a 7-bit binary tuple to represent the relative solvent accessibility features. For example, 0 is represented by [0,0,0,0,0,0,0]^T^ , 1 is represented by [0,1,0,0,0,0,0]^T^, and 6 is represented by [0,0,0,0,0,0,1]^T^.

For all the over-represented or under-represented functional features detected by the Hypergeometric test, if the candidate has the feature it was represented by 1, otherwise it was assigned 0.

### Training and testing with Support Vector Machines

SVMs were used to evaluate the effects of different kinds of features. High sequence similarity between training and testing sets may cause a bias, so we discarded highly homologous sequences (over 70% identity) in the positive dataset before cross-validation to reduce this bias. After removing the highly homologous sequences, sample size for each kinase family was listed in Table 1. It is hard to construct a precise negative phosphorylation site set. Here the negative dataset was randomly selected from the background set with the same sample size as the positive training set. To avoid high sequence similarity in the negative set, during the random selection process, if the selected sequence was over 70% identical with the previous selected sequences, it was removed. The process went on until the required sample size was achieved. To evaluate the prediction performance, a five-fold cross-validation was used in this study. In this process, 4/5 randomly chosen positive samples were used as the training set and the remaining 1/5 were used as the test set. The five-fold cross-validation tests were performed 1000 times and the final evaluation was based on average of these 1000 performances. Since the number of functional features was huge (more than 20 thousand) and the sample size was very small (around 100), only the over-represented or under-represented functional features determined by the hypergeometric tests were used to train the binary classifiers. To avoid selection bias, the set of significant features were re-selected based on samples only in the training set in each cross-validation round.

**Table 1:** The number of known phosphorylation site for different kinase families.

Kinase family	#Known phosphorylation site
ATM (Ataxia telangiectasia mutated)	55
CDK (Cyclin-dependent kinases)	237
CK2 (Casein kinases 2)	206
GSK-3 (Glycogen synthase kinases 3)	53
MAPK (Mitogen-activated protein kinases)	211
PKA (cAMP-dependent protein kinase)	210
PKB (Protein kinases B)	74
PKC (Protein kinase C)	259

To evaluate the effect of various kinds of functional features separately, we constructed different feature groups by adding one functional feature at a time to the sequence features; and also considered combined effects by including them all. The feature groups used in evaluation were: 1) sequence and structure features only; 2) sequence, structure plus significant KEGG features; 3) sequence, structure plus significant GO Biological Process features; 4) sequence, structure plus significant GO Cellular Component features; 5) sequence, structure plus significant GO Molecular Function features; 6) sequence, structure plus significant Pfam domain features; 7) sequence, structure plus significant InterPro domain features; 8) sequence, structure plus significant STRING PPI features; 9) sequence, structure plus the combination of all functional features. So for each round of five-fold cross-validation, nine SVM training and testing processes were performed one at a time. The libSVM [27] package was used here for an individual kinase family with the radial basis function kernel.

## Results

### Scoring proteins with over/under-represented functional features

Using the hypergeometric test, we found over-represented and under-represented functional features for different kinase families. Take the substrates of the CDK family as an example, we found that the CDK substrates are enriched in the Biological Processes “cell cycle”, “cell division”, “mitosis” and “cell proliferation”. Consistent with this result, it is well known that the CDK kinase family is a primary regulator of the cell cycle [15], [16]. The CDK substrates are also found to be enriched in the Cellular Components “cytoplasm” and “transcription factor complex”. Most of the significant Cellular Components are over-represented, except for “integral to membrane” which is under-represented. It indicated that the CDK substrates may less likely to be located in the membrane. For the PPI networks, 964 proteins are found in the over-represented sub-networks that interact with the CDK substrates. The top 10 ranked ones were CDK1, CCNA2, CCNB2, CDK2, CCND3, CCNB3, CCNA1, CCNG1, PCNA and AURKA. For the functional domain data, no enriched domains were found from InterPro, and three functional domains from Pfam were found to be enriched for CDK kinase substrates.

**Figure 1:**
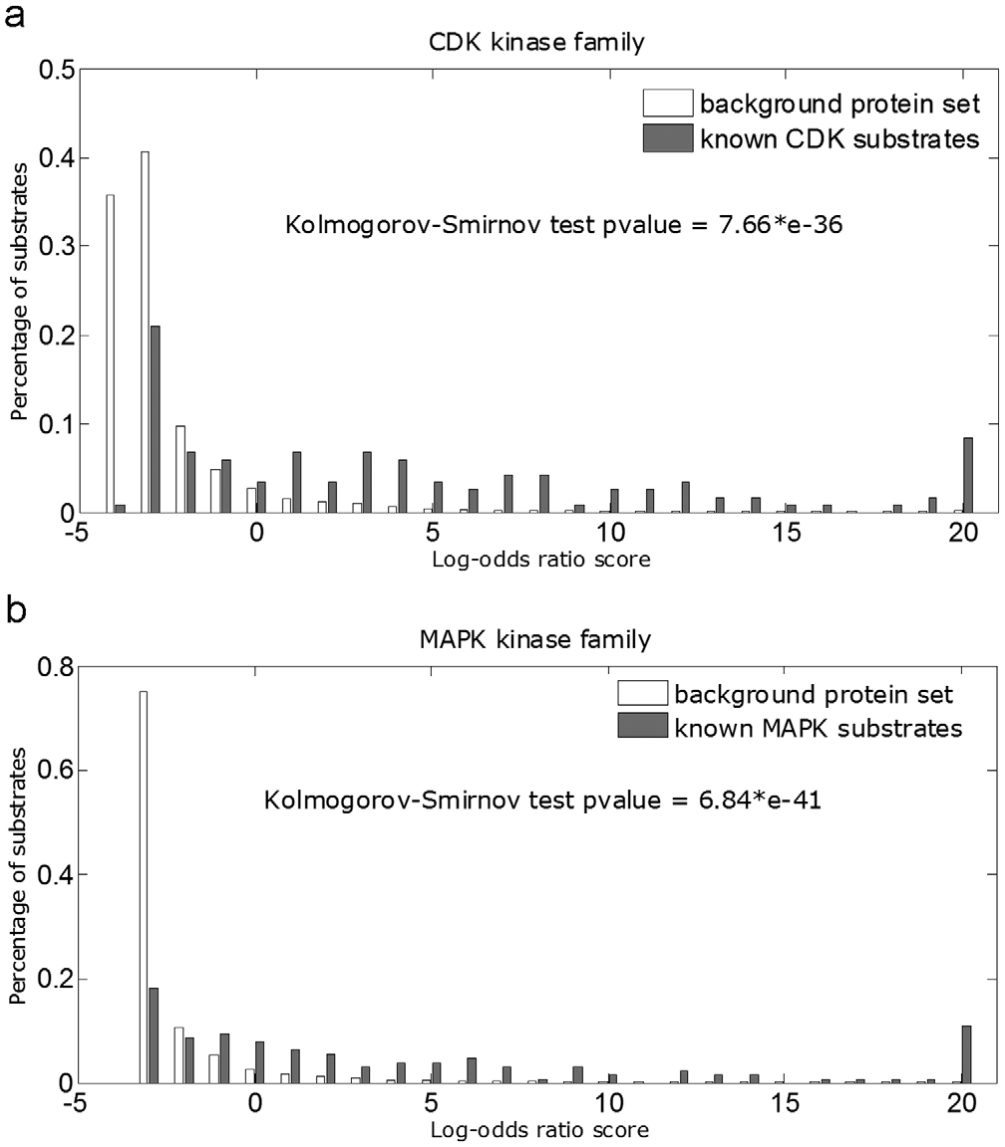
Background protein set (white) and known phosphorylation substrate (grey) score distributions for a) CDK and b) MAPK kinase families. The horizontal axis is the log-odds ratio score and the vertical axis is the percentage of proteins with corresponding scores.

We evaluate the discriminative power of significant functional features using a simple log-odds ratio approach. For each protein, log odds of observing each feature in the phosphorylated over non- phosphorylated class is summed up. The higher the score is, the more likely the protein can be phosphorylated. With this strategy, we scored both the background protein set and the known phosphorylated proteins. We find that score distributions are significantly different for phosphorylated and background proteins across all the kinase families. As examples, the score distributions for the CDK and MAPK kinase families are shown in Figure 1. Most of the proteins in the background protein set (shown as white in the figure) have scores less than 0, while most known phosphorylated proteins (shown in grey) have positive scores. The Kolmogorov-Smirnov test suggested the difference is significant for these two families (p = 7.66*e-36 and 6.84*e-41, respectively). The distributions for the remaining six families can be found in Supplementary Figure S1, with p-values of 8.07*e-19 for ATM, 1.59*e-28 for CK2, 4.04*e-15 for GSK3, 1.04*e-36 for PKA, 8.44*e-25 for PKB and 6.64*e-44 for PKC. These results indicate that the functional features were informative for distinguishing phosphorylated and non-phosphorylated proteins.

### Overall influence of functional features in classification performance

**Figure 2:**
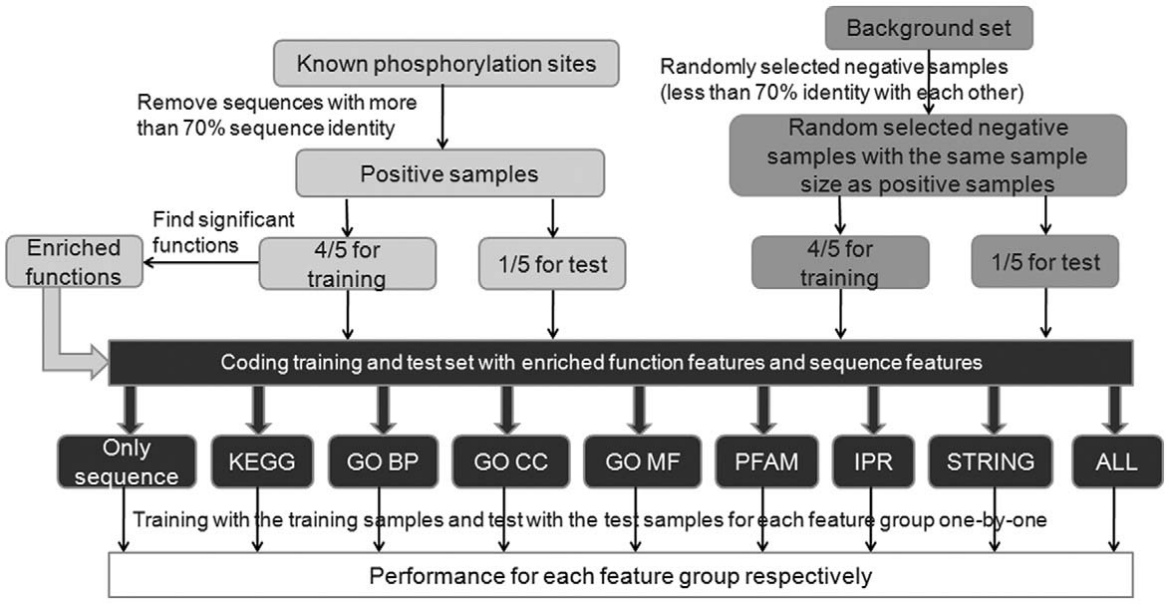
Workflow of the cross-validation test for each kinase family. Before cross-validation, known phosphorylation sequences with higher than 70% sequence identity are removed. Then 4/5 of the positive samples are used as training data and the remaning 1/5 as testing data. Over-represented or under-represented functional features for the substrates of each kinase are got by hypergeometric distributions only based on the training data. The negative samples were randomly selected from the background set. To avoid high sequence similarities in the negative set, in the random selection process if the selected sequence has over 70% sequence identity with the previous selected sequences, it will be removed. The negative sample sizes were the same as the positive sample sizes and the proportion of the training and testing sets were still 4/5 and 1/5. Finally, for the same sample sets, different feature groups were integrated together and trained/tested one at a time. Here “sequence” represents sequence and structure features; “KEGG” represents sequence, structure and significant KEGG features; “GO BP” represents sequence, structure and significant GO Biological Process features; “GO CC” represents sequence, structure and significant GO Cellular Component features; “GO MF” represents sequence, structure and significant GO Molecular Function features; “PFAM” represents sequence, structure and significant Pfam domain features; “IPR” represents sequence, structure and significant InterPro domain features; “STRING” represents sequence, structure and significant STRING PPI features; “ALL” represents an integration of all the above features.

In the performance evaluation, an ideal solution to perform an unbiased comparison is by running cross-validations on all methods using the same dataset. SVMs [28] are an efficient algorithm for solving two-class classification problems in high-dimensional spaces and has been successfully applied in phosphorylation site prediction [7]. Here we try to evaluate the overall influence of functional features on classification performance by SVMs. The training and testing workflow was displayed in Figure 2. In this process, all the SVM classifiers were constructed under the same conditions except that different feature groups were used.

The results of the cross-validation for each kind of functional group were listed in Table 2. The size of negative sample set was the same as the positive sample; the prediction accuracy in Table 2 was simply the percentage of correct predictions among both positive and negative samples. We refer this single value as the prediction accuracy below, since it is easy to compare. We used the gain of prediction accuracy as the measure of the power to include additional functional features. When ranked by the increase of prediction accuracy when adding all functional features (the “minus” column in Table 2) we can see that functional features are most powerful for the GSK3 kinase family. When the effects of each functional group are checked in detail we see that the Cellular Component features from the GO database contribute most to the improvement. For the PKC kinase family, which also has about ten percent increase in accuracy, the protein-protein interaction information from the STRING database are the most powerful. For the CK2 family, the Cellular Component and Molecular Function features from the GO database, and the PPI information from the STRING database seem to all be useful in the performance enhancement. However, the functional features are not effective for every kinase familie. For the ATM kinase family, the performance dropped by about 1 percent.

**Table 2:** Prediction performance of different feature groups by dividing phosphorylation sites.

Kinase	all	sequence	minus	kegg	bp	cc	mf	ipr	pfam	string
GSK3	87.44	76.58	10.86	81.65	79.10	88.77	79.41	76.58	77.20	81.17
PKC	88.08	78.29	9.79	80.93	79.14	81.82	80.12	78.29	78.52	86.18
CK2	87.69	82.42	5.27	84.36	82.42	84.69	85.12	82.42	82.42	85.67
MAPK	92.93	89.10	3.83	91.70	90.17	89.79	89.19	89.10	89.07	91.04
PKA	90.86	89.00	1.86	89.41	89.04	89.53	89.07	89.00	88.98	89.83
PKB	91.90	90.60	1.30	93.19	91.17	90.89	90.47	90.59	90.56	90.35
CDK	94.96	93.68	1.28	94.35	94.42	94.54	93.65	93.68	93.68	94.61
ATM	95.97	97.27	−1.30	97.09	96.73	96.84	97.16	97.27	97.24	95.92

The prediction accuracy was simply the percentage of correct predictions among both positive and negative samples. Here “all” means sequence, structure plus the combination of all functional features; “sequence” means sequence and structure features only; “minus” equals to “all” minus “sequence”; “kegg” means sequence, structure plus significant KEGG features; “bp” means sequence, structure plus significant GO Biological Process features; “cc” means sequence, structure plus significant GO Cellular Component features; “mf” means sequence, structure plus significant GO Molecular Function features; “ipr” means sequence, structure plus significant InterPro domain features; “pfam” means sequence, structure plus significant Pfam domain features; “string” means sequence, structure plus significant STRING PPI features.

The known phosphorylation sites were limited and some of them are on the same protein. If two sites on the same protein are divided into the training and testing sets, this may cause over-estimate for the effect of the functional features. So we redid the above experiments by dividing the known phosphorylated proteins into training and testing sets first and then extracting their known phosphorylation sites as the training and testing samples. With this strategy, the five-fold cross-validation was also performed 1000 times. The final experiment results are listed in Table 3. Though the enhancement in prediction accuracy weakens, it was still significant for some kinase families.

**Table 3:** Prediction performance of different feature groups by dividing phosphorylated proteins.

Kinase	all	sequence	minus	kegg	bp	cc	mf	ipr	pfam	string
gsk3	83.10	77.69	5.42	80.30	77.85	87.24	78.52	77.69	77.68	78.99
pkc	83.65	78.26	5.39	79.81	78.61	81.50	79.04	78.26	78.36	83.46
ck2	84.70	82.43	2.26	83.34	82.10	84.14	84.29	82.43	82.43	83.83
mapk	90.45	89.24	1.21	91.27	89.38	89.91	89.02	89.23	89.03	90.56
cdk	93.99	93.58	0.41	93.93	93.73	94.30	93.53	93.58	93.58	94.24
pka	89.13	88.75	0.37	89.09	88.66	89.02	88.73	88.75	88.71	88.80
pkb	90.89	91.16	−0.27	92.92	91.18	90.97	90.75	91.15	91.13	89.54
atm	92.01	97.55	−5.54	97.46	96.85	96.84	97.35	97.55	97.46	94.45

The meaning of values and shortened forms are the same as those in Table 2.

From the results of Table 2 and Table 3, we can see that the importance of functional features is different for various kinase families. For the GSK3 family, which has the most enhancement, the Cellular Component in GO was the most informative. Checking the enriched cellular component functions of GSK3, we found that six GO terms are enriched for the substrates of the GSK3 kinase family: GO:0005737 (cytoplasm), GO:0005654 (nucleoplasm), GO:0030424 (axon), GO:0005667 (transcription factor complex), GO:0005829 (cytosol) and GO:0005634 (nucleus). For the PKC family, the PPI information in the STRING database was the most informative. For the CK2, MAPK, CDK, PKA and PKB families, no single feature predominate the contribution.

For the ATM family, adding the functional features resulted in a worse performance. From the results of different functional groups, we can see that the adding of KEGG, BP, CC, MF, IPR and PFAM have little influence on the performance; poorer performance was mainly caused by adding the STRING information. Similarly, for the PKB family, the poorer performance also resulted from adding the STRING information. From the results of Figure 1 and Supplementary Figure S1 we can see that, as with the other six kinase families, both the ATM and PKB kinase families can be discriminated via their phosphorylation status based on functional features. Then why do these two families have worse performances after adding functional features? A common characteristic for these two kinase families is that both of them have smaller sample size (Table 1). The poorer performances for both ATM and PKB are caused by the inclusion of STRING information which typically has larger number of features than those in other feature groups. The small sample size with large number of features might cause over-fitting and further bad performance on the test set. With the availability of more phosphorylation training data, we believe the performance for these two kinase families should be as good as the other kinase families. The GSK3 kinase family also has a very small sample size. Its good performance may be explained by the fact that the sample set is easily separated into two classes.

### Identifying kinase-specific phosphorylation sites by integrating multi-functional information

From the above analysis we find that functional features are useful for most of the kinase families. In Figure 1, we can also see that some proteins in the background set have a higher score than the known phosphorylation substrates. These proteins are very similar to the known substrates based on features such as pathways, cellular components and PPI information, so they should have a higher probability to be phosphorylated. To get a set of more accurate candidates, we selected the proteins whose scores are higher than the median of the scores of known substrates. The results in Table 2 and Table 3 demonstrated that SVM classifiers trained by functional features are powerful for the CDK, CK2, GSK3, MAPK, PKA and PKC kinase families. We then scan their possible phosphorylation sites in the candidate set by the SVM classifiers trained using all the known phosphorylation sites, and a negative dataset with the same sample size selected from the background set for these kinase families. The putative phosphorylation sites for CDK, CK2, GSK3, MAPK, PKA and PKC kinase families are avaiable at http://cmbi.bjmu.edu.cn/huphospho, and the corresponding summary are listed in Table 4. Based on the prediction results, the CDK substrates have the highest phosphorylation site density; on average one CDK substrate has nearly five phosphorylation sites. It has been found that many CDK substrates contain multiple clustered phosphorylated sites. This characterisitc had been successfully used in the identification of CDK phosphorylation sites in *Saccharomyces cerevisiae* [29].

**Table 4:** Summary of putative kinase-specific human phosphorylation sites.

Kinase family	Number of candidate sites	Number of predicted phosphorylated proteins	Number of predicted phosphorylation sites
CDK	47787	482	2357
CK2	112246	825	2209
GSK3	47499	104	178
MAPK	71194	113	174
PKA	80764	770	2499
PKC	78895	85	102

## Discussion

The aim of this study was to evaluate the contribution of functional features in phosphorylation site prediction. Protein phosphorylation is a dynamic process which plays key roles in many cellular processes. It is clear that the recognition of phosphorylation sites should be related to many functional features. In the previous studies, the prediction of phosphorylation sites was mainly based on sequence features. Many researchers have pointed out that the cellular component may contain additional information for phosphorylation site prediction, since the phosphorylation cannot happen if the kinase and its putative substrate are not in the same component. The results in this study support this point in that the Cellular Component features are a powerful predictor of phosphorylation status for some kinase families. Besides Cellular Component features, the PPI information was also an effective predictor. A protein kinase usually binds both its substrates and ATP in the phosphorylation process, so the PPI information should provide additional information. It is expected that the Molecular Function and Biological Process should be more powerful for phosphorylation site prediction, since some known phosphorylation proteins may be annotated by the GO terms related to phosphorylation. But the final result indicated that these two are not so powerful for all the kinase families. This eliminates the possibility that the enhancement of functional features is caused by known phosphorylation related annotations of the test samples.

This work demonstrates that prediction of phosphorylation site can be more accurate for most kinase families if we incorporate more biological knowledge in the classification model. Such biological knowledge includes, for example, whether the candidate and the corresponding kinase interact with each other which can be obtained by experiments such as immune-precipitation and the yeast two hybrid systems. It is also informative as *a priori* if a protein can be phosphorylated at all which identified by a common phosphorylation antibody or mass-spectroscopy. All these and other types of biological knowledge, when properly coded into a classification model, are promised to further enhance the prediction performance. Besides phosphorylation, all other kinds of post-translational modifications are functionally related, so our strategy should also be extended to predict other kinds of post-translational modification status.

## Supporting Information

Figure S1 Background protein set (white) and known phosphorylation substrate (grey) score distributions for ATM, CK2, GSK3, PKA, PKB and PKC kinase families. The horizontal axis is the log‐odds ratio score and the vertical axis is the percentage of proteins with corresponding scores. (DOCX)

## References

[1] Manning G, Whyte DB, Martinez R, Hunter T, Sudarsanam S (2002). The protein kinase complement of the human genome.. Science.

[2] Ubersax JA, Ferrell JE (2007). Mechanisms of specificity in protein phosphorylation.. Nat Rev Mol Cell Biol.

[3] Pinna LA, Ruzzene M (1996). How do protein kinases recognize their substrates?. Biochim Biophys Acta.

[4] Kreegipuu A, Blom N, Brunak S, Jarv J (1998). Statistical analysis of protein kinase specificity determinants.. FEBS Lett.

[5] Blom N, Gammeltoft S, Brunak S (1999). Sequence and structure-based prediction of eukaryotic protein phosphorylation sites.. J Mol Biol.

[6] Iakoucheva LM, Radivojac P, Brown CJ, O'Connor TR, Sikes JG (2004). The importance of intrinsic disorder for protein phosphorylation.. Nucleic Acids Res.

[7] Kim JH, Lee J, Oh B, Kimm K, Koh I (2004). Prediction of phosphorylation sites using SVMs.. Bioinformatics.

[8] Dang TH, Van Leemput K, Verschoren A, Laukens K (2008). Prediction of kinase-specific phosphorylation sites using conditional random fields.. Bioinformatics.

[9] Li T, Li F, Zhang X (2008). Prediction of kinase-specific phosphorylation sites with sequence features by a log-odds ratio approach.. Proteins.

[10] Akamine P, Madhusudan, Wu J, Xuong NH, Ten Eyck LF (2003). Dynamic features of cAMP-dependent protein kinase revealed by apoenzyme crystal structure.. J Mol Biol.

[11] Sharrocks AD, Yang SH, Galanis A (2000). Docking domains and substrate-specificity determination for MAP kinases.. Trends Biochem Sci.

[12] Pawson T, Scott JD (1997). Signaling through scaffold, anchoring, and adaptor proteins.. Science.

[13] Gnad F, Ren S, Cox J, Olsen JV, Macek B (2007). PHOSIDA (phosphorylation site database): management, structural and evolutionary investigation, and prediction of phosphosites.. Genome Biol.

[14] Linding R, Jensen LJ, Ostheimer GJ, van Vugt MA, Jorgensen C (2007). Systematic discovery of in vivo phosphorylation networks.. Cell.

[15] Stillman B (1996). Cell cycle control of DNA replication.. Science.

[16] Zachariae W, Nasmyth K (1999). Whose end is destruction: cell division and the anaphase-promoting complex.. Genes Dev.

[17] Andres V (2004). Control of vascular cell proliferation and migration by cyclin-dependent kinase signalling: new perspectives and therapeutic potential.. Cardiovasc Res.

[18] Yao S, Prelich G (2002). Activation of the Bur1-Bur2 cyclin-dependent kinase complex by Cak1.. Mol Cell Biol.

[19] Oelgeschlager T (2002). Regulation of RNA polymerase II activity by CTD phosphorylation and cell cycle control.. J Cell Physiol.

[20] Blom N, Sicheritz-Ponten T, Gupta R, Gammeltoft S, Brunak S (2004). Prediction of post-translational glycosylation and phosphorylation of proteins from the amino acid sequence.. Proteomics.

[21] Diella F, Cameron S, Gemund C, Linding R, Via A (2004). Phospho.ELM: a database of experimentally verified phosphorylation sites in eukaryotic proteins.. BMC Bioinformatics.

[22] Diella F, Gould CM, Chica C, Via A, Gibson TJ (2008). Phospho.ELM: a database of phosphorylation sites–update 2008.. Nucleic Acids Res.

[23] Yaffe MB, Leparc GG, Lai J, Obata T, Volinia S (2001). A motif-based profile scanning approach for genome-wide prediction of signaling pathways.. Nat Biotechnol.

[24] Xue Y, Ren J, Gao X, Jin C, Wen L (2008). GPS 2.0, a tool to predict kinase-specific phosphorylation sites in hierarchy.. Mol Cell Proteomics.

[25] Wagner M, Adamczak R, Porollo A, Meller J (2005). Linear regression models for solvent accessibility prediction in proteins.. J Comput Biol.

[26] Team RDC (2010).

[27] Lin C-CCaC-J (2001).

[28] Vapnik VN (1999). An overview of statistical learning theory.. IEEE Trans Neural Netw.

[29] Chang EJ, Begum R, Chait BT, Gaasterland T (2007). Prediction of cyclin-dependent kinase phosphorylation substrates.. PLoS One.

