# Supplementary material for: Identifying Human Kinase-Specific Protein Phosphorylation Sites by Integrating Heterogeneous Information from Various Sources
Source: PLoS One. 2010 Nov 15;5(11):e15411. doi: 10.1371/journal.pone.0015411 (PMC2981550; doi:10.1371/journal.pone.0015411)
Supplement: Figure S1 — Background protein set (white) and known phosphorylation substrate (grey) score distributions for ATM, CK2, GSK3, PKA, PKB and PKC kinase families. The horizontal axis is the log‐odds ratio score and the vertical axis is the percentage of proteins with corresponding scores. (DOCX) [file pone.0015411.s001.docx]

Figure S1. Background protein set (white) and known phosphorylation substrate (grey) score distributions for ATM, CK2, GSK3, PKA, PKB and PKC kinase families. The horizontal axis is the log-odds ratio score and the vertical axis is the percentage of proteins with corresponding scores.
